# Effect of Post-mortem Interval and Perfusion on the Biophysical Properties of *ex vivo* Liver Tissue Investigated Longitudinally by MRE and DWI

**DOI:** 10.3389/fphys.2021.696304

**Published:** 2021-08-03

**Authors:** Karolina Garczyńska, Heiko Tzschätzsch, Sanam Assili, Anja A. Kühl, Akvile Häckel, Eyk Schellenberger, Nikolaus Berndt, Hermann-Georg Holzhütter, Jürgen Braun, Ingolf Sack, Jing Guo

**Affiliations:** ^1^Department of Radiology, Berlin Institute of Health, Charité - Universitätsmedizin Berlin, Corporate Member of Freie Universität Berlin, Humboldt-Universität zu Berlin, Berlin, Germany; ^2^Department of Veterinary Pathology, College of Veterinary Medicine, Freie Universität Berlin, Berlin, Germany; ^3^Department of Biology, SUNY Albany, Albany, NY, United States; ^4^iPATH.Berlin - Core Unit of Charité, Universitätsmedizin Berlin, Corporate Member of Freie Universität Berlin, Humboldt-Universität zu Berlin, Berlin, Germany; ^5^Institute for Imaging Science and Computational Modelling in Cardiovascular Medicine, Berlin Institute of Health (BIH), Charité - Universitätsmedizin Berlin, Corporate Member of Freie Universität Berlin, Humboldt-Universität zu Berlin, Berlin, Germany; ^6^Computational Systems Biochemistry Group, Institute of Biochemistry, Berlin Institute of Health (BIH), Charité - Universitätsmedizin Berlin, Corporate Member of Freie Universität Berlin, Humboldt-Universität zu Berlin, Berlin, Germany; ^7^Institute of Medical Informatics, Berlin Institute of Health, Charité - Universitätsmedizin Berlin, Corporate Member of Freie Universität Berlin, Humboldt-Universität zu Berlin, Berlin, Germany

**Keywords:** liver stiffness, viscosity, perfusion, degradation, magnetic resonance elastography (MRE), diffusion-weighted imaging (DWI), water diffusivity, post-mortem interval

## Abstract

Structural changes of soft tissues on the cellular level can be characterized by histopathology, but not longitudinally in the same tissue. Alterations of cellular structures and tissue matrix are associated with changes in biophysical properties which can be monitored longitudinally by quantitative diffusion-weighted imaging (DWI) and magnetic resonance elastography (MRE). In this work, DWI and MRE examinations were performed in a 0.5-Tesla compact scanner to investigate longitudinal changes in water diffusivity, stiffness and viscosity of ex-vivo rat livers for up to 20 h post-mortem (pm). The effect of blood on biophysical parameters was examined in 13 non-perfused livers (containing blood, NPLs) and 14 perfused livers (blood washed out, PLs). Changes in cell shape, cell packing and cell wall integrity were characterized histologically. In all acquisitions, NPLs presented with higher shear-wave speed *(c)*, higher shear-wave penetration rate *(a)* and smaller apparent-diffusion-coefficients (ADCs) than PL. Time-resolved analysis revealed three distinct phases: (i) an initial phase (up to 2 h pm) with markedly increased c and a and reduced ADCs; (ii) an extended phase with relatively stable values; and (iii) a degradation phase characterized by significant increases in *a* (10 h pm in NPLs and PLs) and ADCs (10 h pm in NPLs, 13 h pm in PLs). Histology revealed changes in cell shape and packing along with decreased cell wall integrity, indicating tissue degradation in NPLs and PLs 10 h pm. Taken together, our results demonstrate that the biophysical properties of fresh liver tissue rapidly change within 2 h pm, which seems to be an effect of both cytotoxic edema and vascular blood content. Several hours later, disruption of cell walls resulted in higher water diffusivity and wave penetration. These results reveal the individual contributions of vascular components and cellular integrity to liver elastography and provide a biophysical, imaging-based fingerprint of liver tissue degradation.

## Introduction

Changes in cellular and extracellular matrix (ECM) architecture associated with pathological or physiological processes affect the biophysical tissue properties that are visualized by medical imaging. However, the exact link between microarchitectural changes and the corresponding biophysical manifestations on the macroscopic imaging level is still elusive. Especially for the liver, where imaging of viscoelastic tissue properties by elastography has become a standard clinical procedure, a deeper understanding of the structure elements that determine the changes in macroscopic imaging properties is urgently needed (Mueller, 2020; Hudert et al., 2021).

Changes of the tissue’s microarchitecture can be caused by either pathological factors (such as inflammation, fibrosis, etc.) or physiological factors (such as post-mortem cellular apoptosis, blood perfusion status, etc.). Post-mortem (pm) degeneration of biological tissues is a process that involves alterations of tissue composition and structure on many levels. The degree of *ex vivo* decomposition is determined by many factors, including the tissue type, post-mortem interval, temperature, and humidity (Oka, 1920; Otto et al., 1981; Milroy, 1999; Tomita et al., 2004). Earlier studies investigating the time course of tissue decomposition from biochemical and morphological perspectives have revealed changes in macro- and microstructures (Benda et al., 1957; Masshoff et al., 1964; Riede et al., 1976), glycogen concentration (Popper and Wozasek, 1932; Hertz, 1933; Nunley et al., 1973; Calder and Geddes, 1990), pH level and fat content (Shima, 1922; Sinapius, 1963; Donaldson and Lamont, 2013). In view of the structural and functional changes involved in the post-mortem degeneration process, serial characterization of the biophysical properties of *ex vivo* tissues by imaging with simultaneous histological validation could help us elucidate the link between imaging parameters and microscopic tissue structure.

Magnetic resonance imaging (MRI) has been employed to investigate the morphological and biophysical properties of *ex vivo* tissue specimens (Schneiderman et al., 2005; Tempel-Brami et al., 2015; Nebelung et al., 2016; Wolfram et al., 2020). In the context of tissue degradation, water diffusivity measured by diffusion-weighed imaging (DWI) has been revealed as a potential marker of post-mortem tissue alterations (Arthurs et al., 2015; Keller et al., 2018; Sapienza et al., 2019). Three studies investigating *ex vivo* brain tissue *in situ* have also demonstrated biomechanical properties quantified by magnetic resonance elastography (MRE) to be sensitive to instantaneous cerebral structural and functional changes occurring after death (Vappou et al., 2008; Weickenmeier et al., 2018; Bertalan et al., 2020). Although published studies provide imaging data on *ex vivo* tissue, most publications report results obtained at a single point in time. To our knowledge, data from systematic MRI studies of tissue without fixation over a long post-mortem interval (>10 h) are very limited (Keller et al., 2018; Sapienza et al., 2019). The recently introduced compact MRI device with MRE capability is an excellent tool for performing repeated examinations for the longitudinal investigation of *ex vivo* tissue samples (Braun et al., 2018; de Schellenberger et al., 2019; Sauer et al., 2019; Everwien et al., 2020; Garczyńska et al., 2020). The cylindrical sample tube utilized by this device ensures well-defined boundary conditions, which is beneficial for MRE analysis, and this technique has been shown to provide consistent values of stiffness and viscosity (Ipek-Ugay et al., 2015; Braun et al., 2018; Garczyńska et al., 2020).

In the present study, we used this compact MRI/MRE device to longitudinally investigate changes in the biophysical properties of *ex vivo* liver samples harvested from rats in a post-mortem interval of up to 20 h. In addition to the effect of the duration of the pm interval, we investigated the possible effect of the presence of blood on tissue decomposition by comparing the biophysical features of perfused and non-perfused liver samples. This was done because tissue perfusion is a common sample preparation step for histopathological analysis in science, especially for immunohistology (Scouten et al., 2006). Moreover, our imaging findings were supported by histological findings. Our foremost aim using this setup was to further the understanding of the underlying microscopic structural changes that manifest as macroscopic imaging features. Specifically, we quantified the apparent water diffusion coefficient (ADC) by DWI as well as stiffness-related shear wave speed (*c*) and viscosity-related shear wave penetration rate (*a*) by MRE in order to provide a specific fingerprint of the biophysical alterations of perfused and non-perfused liver tissue over time after death.

## Materials and Methods

All procedures involving animals were approved by the local authority (Landesamt für Gesundheit und Soziales Berlin, Reg. No. T0212/19) and were performed according to animal welfare regulations and institutional guidelines.

### Sample Preparation

Livers were harvested from young adult female Wistar rats (Forschungseinrichtungen für Experimentelle Medizin, FEM, Berlin, Germany; Janvier Labs, Le Genest-Saint-Isle, France). All rats were kept in the same animal facility under standard housing conditions for at least 3 days before imaging. In total, 41 rats were sacrificed and livers were harvested and randomly selected for two groups: (i) non-perfused liver (NPL, n_total = 20 with *n* = 13 for the MRE/DWI investigations and *n* = 7 for histology) and (ii) perfused liver (PL, n_total = 21 with *n* = 14 for the MRE/DWI investigations and *n* = 7 for histology).

To harvest the livers, the rats were first anesthetized with an overdose of isoflurane vapor. For the NPL group, the rats were decapitated with a rodent guillotine and the livers were removed without further processing. For the PL group, anesthesia was followed by exsanguinations, and the liver was perfused *in situ*. For liver perfusion, the portal vein (PV) was cannulated (20G Vasofix Safety IV Catheter, B. Braun, Melsungen, Germany) and the inferior vena cava (IVC) was severed and used as outflow. Blood was washed out using a peristaltic pump (MEDOREX TBE/200 62-4-3-3, MDX Biotechnik International, Nörten-Hardenberg, Germany), and circulating cells were removed from the liver by phosphate-buffered saline solution (PBS Tablets, Gibco, Thermo Fisher Scientific, Paisley, United Kingdom). Perfusion was stopped upon the observation of a non-decoloring liver with clear outflow. Average perfusion time was 13 min at a perfusion rate of approx. 40 ml/min and a total perfusate consumption of ~700 ml.

For MR imaging, a cylindrical sample (8 mm Ø, 20 mm height) was cut out from the left lateral lobe of the liver and directly transferred into a glass tube, which was then inserted into a compact MRI device (Pure Devices GmbH, Würzburg, Germany) for imaging. Sample preparation time was 19 min for NPLs and 32 min for PLs (13 min extra for perfusion). As a result, MRI started 19 min pm for NPLs and 32 min pm for PLs.

### Magnetic Resonance Elastography (MRE) and Diffusion-Weighted Imaging (DWI)

All imaging examinations were performed in a 0.5-T compact MRI device as mentioned above. For MRE measurements, an external gradient amplifier (DC 600, Pure Devices GmbH, Würzburg, Germany) and a piezo-actuator (PAHL60/20 Piezosystem Jena, Jena, Germany) coupled to the sample tube were implemented to the MRI device to provide mechanical vibrations. Details of the MRE setup are provided in Braun et al. (2018); de Schellenberger et al. (2019); Sauer et al. (2019); Everwien et al. (2020) and Garczyńska et al. (2020).

Imaging parameters for MRE and DWI were the same as described in Garczyńska et al. (2020). For MRE, one axial 3 mm slice with an in-plane resolution of 150 μm was acquired using a mechanical driving frequency of 800 Hz. The tissue deflection generated by the mechanical vibration was 0.67 μm. Eight time steps were recorded over a wave cycle within an acquisition time of 3 min. For DWI, the same slice with an in-plane resolution of 600 μm was acquired using seven b-values (50, 175, 300, 425, 550, 675, and 800 s/mm2), which took a total of 5 min. Data from MRE and DWI were repeatedly acquired in an interleaved manner up to 15 h pm. MRE was discontinued thereafter due to visible liquid accumulation between the liver tissue and the sample tube wall, which compromised contact and effectively hindered transfer of mechanical vibrations. Conversely, DWI acquisition, which does not require wave propagation, was continued until 20 h after death. To minimize the possible influence of temperature (Bertalan et al., 2019), the sample was kept at a constant temperature of 30°C throughout the imaging process by a built-in temperature control unit of the MRI scanner.

All imaging data were postprocessed using algorithms written in MATLAB (R2019b, The Mathwork Inc., Natick, MA, United States) as described previously in Braun et al. (2018) and de Schellenberger et al. (2019). MRE-based shear wave speed (*c* in m/s) and shear wave penetration rate (*a* in m/s) were derived using a Bessel function fit. *c* is a surrogate marker of tissue stiffness which can be converted to stiffness in kPa, assuming the elastic model, by ρc2 with ρ being the material’s density of 1,000 kg/m^3^. *a* is inversely correlated to viscous damping of shear waves. For DWI, maps of apparent diffusion coefficient (ADC), which quantifies water diffusivity, were generated with mono-exponential fitting using all seven *b*-values.

### Histology

For histology, 14 additional rats (7 per group) were used, and liver tissue specimens from the left lateral lobe were investigated at three time points: fresh (immediately after death for NPLs and 13 min pm for PLs), 2 h pm, and 10 h pm. To ensure that the histologic features examined reflected the same intrinsic tissue properties of specimens subjected to MRE, all specimens assigned to histology were exposed to the same imaging protocols, including temperature and mechanical fluctuation. In order to minimize disturbance, each liver sample was divided into two portions separated in the sample tube by a small piece of foam earplug. After 1.5 h of MRI acquisition (2 h pm), the top liver portion was removed and prepared for histology while the bottom liver portion remained in the sample tube for another 8 h of MRI examinations and was finally removed for histology at 10 h pm. As the liver samples for histology were divided into 2 portions and were only imaged up to 10 h, the imaging data were not included in the final data analysis.

Tissue samples from all 3 time points were fixed in 4 % formaldehyde solution (Formalin Solution 4 %, J.T. Baker, Fisher Scientific, Avantor, Gliwice, Poland) at room temperature for 24 h. The fixed samples were processed as described earlier (Garczyńska et al., 2020). Paraffin sections of 2 μm thickness were stained with hematoxylin and eosin (H&E; Mayer’s Hemalum Solution, Merck, Darmstadt, Germany; Eosin Y solution, Sigma-Aldrich, Darmstadt, Germany).

Images of stained sections were taken with a BZ-X800 fluorescence microscope (KEYENCE DEUTSCHLAND GmbH, Neu-Isenburg, Germany). For each time point, two high-power fields per sample were analyzed. Hepatocytes were counted per field of view (FoV) at 40x magnification in H&E-stained sections using ImageJ software version 1.52v (Schneider et al., 2012). Histological analysis was performed in a blinded manner.

### Statistical Analysis

Mixed analysis of variance (ANOVA) was performed to account for the effects and interactions of two factors present in our data- acquisition time (within-subject factor) and perfusion status (between-subject factor; non-perfused vs. perfused) for all imaging parameters.

Dynamic responses of water diffusivity over time as quantified by ADC were fitted using the exponential model:

$$ADC=ADC_{0}+\left(ADC_{\text{p}}-ADC_{0}\right)\times \left(1-exp\left(-k\times t\right)\right)$$

where *ADC*_0_ and *ADC*_p_ are initial and plateau ADC values in mm^2^/s, *k* is the rate constant in hours^–1^, and *t* is acquisition time in hours.

Normality of the datasets was first assessed using the D’Agostino-Pearson normality test. Differences between groups were assessed using the unpaired *t*-test for data with normal distribution while the Mann-Whitney test was used for data without normal distribution.

*P*-values below 0.05 were considered statistically significant. Graphical and statistical analysis was performed with GraphPad Prism (GraphPad Prism 8.01. for Windows, GraphPad Software, San Diego, California, United States).^1^

## Results

### General Characterization

**FIGURE 1:**
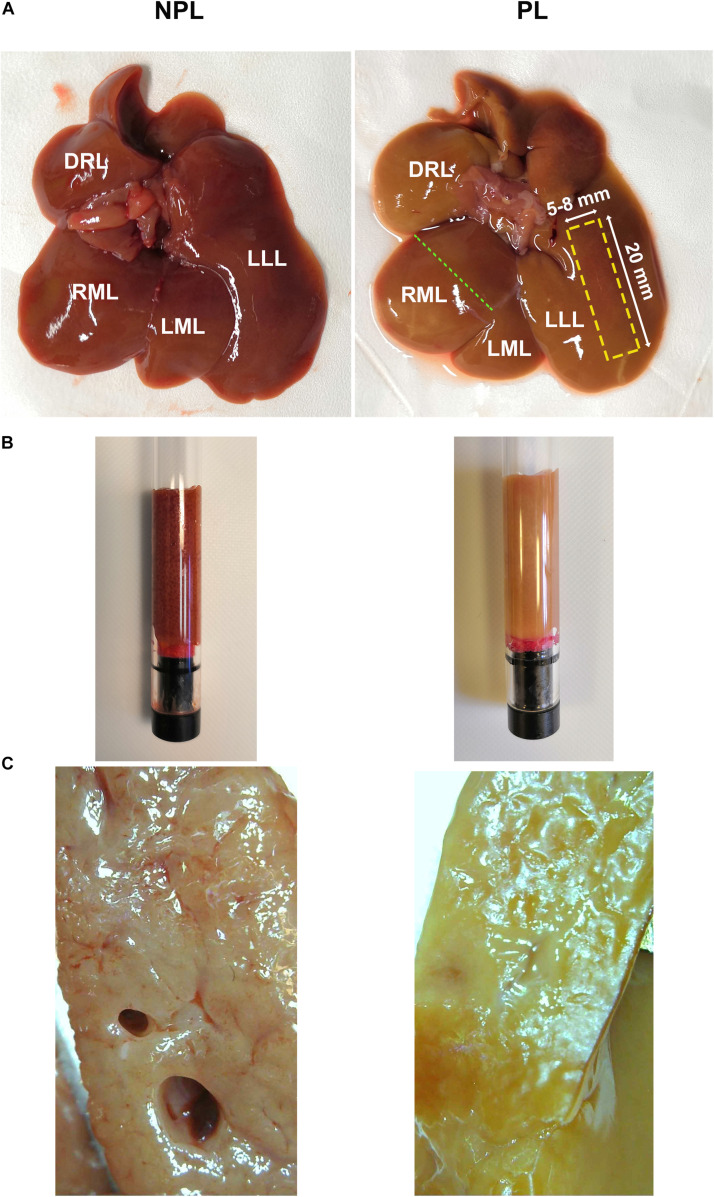
**(A)** Photograph of fresh non-perfused livers (NPLs, left) and perfused livers (PLs, right); a rectangular area marked by a dashed yellow line indicates the site from which the sample for MRI was taken. The green dashed line indicates the position of the cross-sectional area shown in **(C)**. **(B)** Sample tubes containing liver tissue obtained from NPL and PL for DWI and MRE. **(C)** cross-sectional area of the liver showing more blood in the NPL sample (left) than in the PL sample (right). NPL, non-perfused liver; PL, perfused liver; LLL, left lateral lobe; LML, left middle lobe; RML, right middle lobe; DRL, dorsal right lobe.

The difference in appearances between NPL and PL samples is shown in Figure 1. PLs appeared paler and contained less blood than NPLs. Note that, as a result of incomplete perfusion, residual blood was still visible in PLs.

### Magnetic Resonance Elastography

**FIGURE 2:**
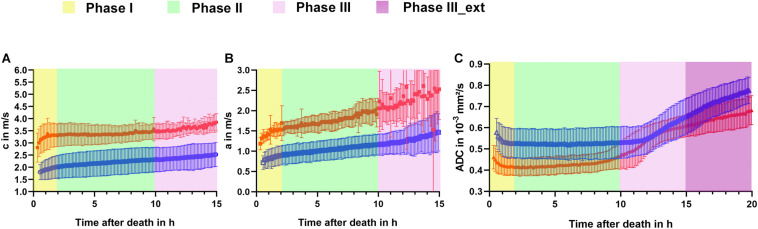
Temporal evolution curves of group averages and standard deviations of **(A)** shear wave speed *c*, **(B)** penetration rate *a*, and **(C)** apparent diffusion coefficient ADC of non-perfused (red curve) and perfused (blue curve) liver with different colors indicating phases as defined in the text. NPL, non-perfused liver; PL, perfused liver.

Time evolution curves of group averages and standard deviations (SDs) of shear wave speed *c* and penetration rate *a* for NPLs and PLs are presented in Figure 2. Both *c* (2.81 ± 0.60 m/s) and *a* (1.19 ± 0.15 m/s) were initially higher in NPLs than PLs (*c*: 1.80 ± 0.31 m/s; *a*: 0.72 ± 0.17 m/s); the difference was 35.94% (*p* ≤ 0.001) and 39.50% (*p* ≤ 0.001), respectively. Based on the development of parameters over time, we identified three distinct phases: Phase I from the start of the first imaging experiment to 2 h pm; Phase II from 2 to 10 h pm; and Phase III from 10 to 15 h pm. In Phase I, an initial increase in both *c* and *a* was observed in both NPLs and PLs. While the increase in *c* did not differ significantly between the two groups, the increase in *a* was more pronounced in the PL group (*c*: 2.88 ± 2.82 %/h for NPLs vs. 4.45 ± 5.13 %/h for PLs, *p* = 0.34; *a*: 4.99 ± 5.99 %/h for NPLs vs. 9.78 ± 3.94 %/h for PLs, *p* ≤ 0.05). In Phase II, both *c* and *a* were relatively stable over time in both groups. For *c*, a significantly higher increase was observed in PLs (NPLs: 0.65 ± 0.86 %/h vs. PLs: 1.58 ± 0.41 %/h, *p* ≤ 0.01). The increase in *a* was similar in both groups with 2.85 ± 1.33 %/h in NPLs and 2.86 ± 0.66 %/h in PLs (*p* = 0.98). In Phase III, both *c* and *a* increased continuously over time without a significant difference between the two groups (*c*: 1.59 ± 1.91 %/h for NPLs vs. 1.59 ± 1.41 %/h for PLs, *p* = 0.99; *a*: 4.09 ± 3.07 % for NPLs vs. 3.75 ± 2.40 %/h for PLs, *p* = 0.75). Interestingly, the SD of *a* increased notably in both NPLs and PLs compared to Phases I and II (SD: NPLs: 0.13 ± 0.07 m/s, PLs: 0.06 ± 0.03 m/s for Phase I vs. NPLs: 0.17 ± 0.05 m/s, PLs: 0.08 ± 0.01 m/s for Phase II vs. NPLs: 0.39 ± 0.31 m/s, PLs: 0.11 ± 0.09 m/s for Phase III, all *p* ≤ 0.001). The rate of parameters variation was calculated by comparing the two values at the beginning and the end of each phase taking the phase duration into consideration. The SD of each phase was obtained by averaging the SD of each measurement point in the corresponding phase. All MRE results are presented in Table 1. The changes in MRE parameters over time are summarized in Table 2.

**TABLE 1:** Summary of variation rates of MRI-based parameters (in %/h) in different post-mortem phases for non-perfused (NPL) and perfused (PL) livers and pairwise comparison of phases.

	Phase I			Phase II			Phase III			Phase III_ext			Comparison of phases	
	NPL	PL	*P*	NPL	PL	*P*	NPL	PL	*P*	NPL	PL	*P*	NPL	PL
***c*** *in %/h*	2.88 ± 2.82	4.45 ± 5.13	0.34	0.65 ± 0.86	1.58 ± 0.41	≤ 0.01	1.59 ± 1.91	1.59 ± 1.41	0.99	/	/	/	P1 vs. P2: *p* ≤ 0.05 P1 vs. P3: *p* = 0.21 P2 vs. P3: *p* = 0.12	P1 vs. P2: *p* ≤ 0.05 P1 vs. P3: *p* = 0.05 P2 vs. P3: *p* = 0.98
***a*** *in %/h*	4.99 ± 5.99	9.78 ± 3.94	≤ 0.05	2.85 ± 1.33	2.86 ± 0.66	0.98	4.09 ± 3.07	3.75 ± 2.40	0.75	/	/	/	P1 vs. P2: *p* = 0.22 P1 vs. P3: *p* = 0.64 P2 vs. P3: *p* = 0.19	P1 vs. P2: *p* ≤ 0.001 P1 vs. P3: *p* ≤ 0.001 P2 vs. P3: *p* = 0.12
***ADC*** *in %/h*	−2.75 ± 1.57	−4.95 ± 2.77	≤ 0.05	1.23 ± 1.17	0.09 ± 0.43	≤ 0.01	4.65 ± 0.94	3.50 ± 1.99	0.07	2.12 ± 0.69	3.26 ± 1.22	≤ 0.01	P1vs. P2: *p* ≤ 0.001 P1 vs. P3: *p* ≤ 0.001 P2 vs. P3: *p* ≤ 0.001 P1 vs. P3_ext: *p* ≤ 0.001 P2 vs. P3_ext: *p* ≤ 0.01 P3 vs. P3_ext: *p* ≤ 0.001	P1 vs. P2: *p* ≤ 0.001 P1 vs. P3: *p* ≤ 0.001 P2 vs. P3: *p* ≤ 0.001 P1 vs. P3_ext: *p* ≤ 0.001 P2 vs. P3_ext: *p* ≤ 0.001 P3 vs. P3_ext: *p* = 0.69

*P1 = Phase I, P2 = Phase II, P3 = Phase III, P3_ext = extended Phase III (DWI).*

**TABLE 2:** Main results of MRE, DWI and histological analysis in both non-perfused liver (NPL) and perfused liver (PL) samples at different phases and time points after death.

Method	Observation/parameter	Phase I		Phase II		Phase III	
		NPL	PL	NPL	PL	NPL	PL

MRE	*c*	↑	↑↑	↑	↑	↑	↑
	*a*	↑↑	↑↑↑	↑	↑	↑↑	↑↑
	ADC	↓	↓↓	↑	↔	↑↑	↑↑

		Fresh		2h post-mortem		10h post-mortem	
Histology	Hepatocyte organization	Tight	Loose	Loose, a few separated cells	Separated cells	Loose, separated cells	Separated cells
	Hepatocyte shape	Polygonal	Round	Round	Round + polygonal	Elongated	Elongated
	Cell wall visibility	High	High	Moderate	Moderate	Low	Moderate
	Glycogen amount	High	Low	Low	None	None	None

*For the imaging parameters, relative changes are qualitatively indicated as increase (↑), decrease (↓), or unchanged (↔). The number of arrows indicates the degree of changes.*

To analyze the influence of time after death and perfusion status on MRE parameters, mixed ANOVA analysis was performed separately for each of the three phases. First, in Phases I and II, the significantly higher values of *c* and *a* in NPLs than in PLs were found to be attributable to the factors time (*p* ≤ 0.001) and perfusion status (*p* ≤ 0.001) with a significant interaction between them (all *p* ≤ 0.01). In Phase III, the two factors (i.e., time and perfusion status) continued to have a significant influence on both *c* and *a*, resulting in higher *c* and *a* values in NPLs than in PLs (all *p* ≤ 0.01). Additionally, these two factors had independent effects on *c* and *a* with no significant interactions between them (*c*: *p* = 0.90; *a*: *p* = 0.88). All mixed ANOVA results are summarized in Table 3.

**TABLE 3:** Summary of mixed model ANOVA results and initial values (means with SDs) in different phases during the acquisition interval in non-perfused liver (NPL) and perfused liver (PL) samples.

	Phase I					Phase II					Phase III					Phase III_ext				
	Initial value		Time	Status	Interaction	Initial value		Time	Status	Interaction	Initial value		Time	Status	Interaction	Initial value		Time	Status	Interaction
	NPL	PL				NPL	PL				NPL	PL				NPL	PL			
***c*** *in m/s*	2.81 ± 0.60	1.80 ± 0.31	***	***	***	3.31 ± 0.45	2.03 ± 0.47	***	***	***	3.31 ± 0.45	2.32 ± 0.50	**	***	ns	/	/	/	/	/
***a*** *in m/s*	1.19 ± 0.15	0.72 ± 0.17	***	***	**	1.53 ± 0.15	0.90 ± 0.24	***	***	***	1.91 ± 0.31	1.16 ± 0.26	***	***	ns	/	/	/	/	/
***ADC*** *in 10^–3^ mm^2^/s*	0.46 ± 0.05	0.58 ± 0.07	***	***	ns	0.42 ± 0.04	0.53 ± 0.07	***	**	***	0.47 ± 0.06	0.53 ± 0.07	***	ns	***	0.61 ± 0.05	0.65 ± 0.08	***	**	***

NPLs (n = 13); PLs (n = 14); ns, non-significant, **p ≤ 0.01, ***p ≤ 0.001.

### Diffusion-Weighted Imaging

As for MRE, we divided the period of DWI acquisition into different phases, based on group averaged apparent diffusion coefficient (ADC) curves over time as shown in Figure 2C. In addition to the three phases defined for MRE, an extended phase III, Phase III_ext, from 15 to 20 h pm was introduced due to the longer DWI acquisition interval. Based on initial ADC values, NPL samples demonstrated 26.08% lower water diffusivity than PL samples (NPLs: 0.46 × 10^–3^ ± 0.05 mm^2^/s vs. PLs: 0.58 × 10^–3^ ± 0.07 mm^2^/s, *p* ≤ 0.001).

Contrary to MRE parameters, ADC values in Phase I decreased in both groups, and the decrease was more pronounced in the PL group (NPLs: −2.75 ± 1.57%/h vs. PLs: −4.95 ± 2.77%/h, *p* ≤ 0.05). In Phase II, a 1.23 ± 1.17%/h increase in ADC was observed in NPLs while the ADC was almost unchanged in PLs (0.09 ± 0.43 %/h, *p* ≤ 0.01). A marked increase in ADC was found for both groups in Phase III and III_ext. While the increase was not significantly different between NPLs and PLs in Phase III (NPLs: 4.65 ± 0.94%/h vs. PLs: 3.50 ± 1.99%/h, *p* = 0.07), PLs exhibited a significantly higher increase than NPLs in Phase_III_ext (NPLs: 2.12 ± 0.69 %/h vs. PLs: 3.26 ± 1.22 %/h, *p* ≤ 0.01). All averaged ADC values are summarized in Table 1. Changes in ADC in the three phases are provided in Table 2.

Mixed ANOVA was also performed to assess the effects of perfusion status and time after death on ADC. In all four phases, time after death had a significant effect on ADC values between the NPL and PL groups (*p* ≤ 0.001) while the effect of perfusion status was only significant in Phases I, II, and III_ext (Phase I: *p* ≤ 0.001; Phases II and III_ext: *p* ≤ 0.01; Phase III: *p* = 0.24). The two factors considered in mixed ANOVA demonstrated significant interactions in all phases (*p* ≤ 0.001) except Phase I (*p* = 0.97). All results of mixed ANOVA are presented in Table 3.

The ADC curves revealed a strong dynamic change during Phase III with different onset time between the NPL and PL groups. To better describe this change, exponential fitting as described in the Methods section was performed for each individual sample from Phase II to Phase III_ext.

**FIGURE 3:**
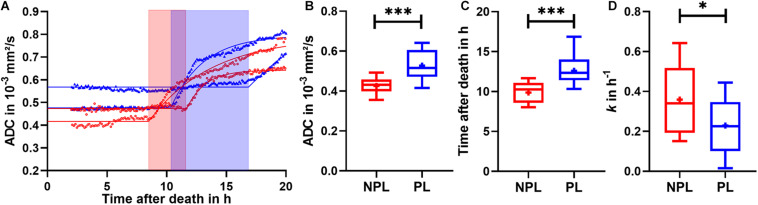
**(A)** ADC data of two samples with the earliest and latest onset of ADC increase have been selected for illustration, separately for NPLs (red) and PLs (blue). Solid lines represent fitted curves using the exponential model. Colored blocks indicate the time duration of the observed ADC increase. Box plots of **(B)** ADC_0_-initial fitted ADC values in mm^2^/s **(C)** onset time of the ADC increase in hour and **(D)** *k*-rate constant in h^− 1^. Data are presented as minimum to maximum with interquartile range and median; + indicates means. ^∗^*p* ≤ 0.05; ^∗∗∗^*p* ≤ 0.001. NPL, non-perfused liver; PL, perfused liver.

In Figure 3A, the fitted curves for samples with the earliest and the latest onset of ADC increase in both NPLs and PLs are selectively shown. Comparison of the two groups showed the initial value, ADC_0_, to be significantly higher in PLs than in NPLs (PLs: 0.53 × 10^–3^ ± 0.07 × 10^–3^ mm^2^/s vs. NPLs: 0.43 × 10^–3^ ± 0.04 × 10^–3^ mm^2^/s, *p* ≤ 0.001, Figure 3B). Secondly, the averaged onset time for the ADC increase in the NPL group was significantly earlier than in the PL group (NPLs: 9.88 ± 1.29 h vs. PLs: 12.59 ± 1.78 h, *p* ≤ 0.001, Figure 3C). Thirdly, rate constant *k*, which denotes the degree of ADC increase over time, was also significantly different between the NPL and PL groups (NPLs: 0.36 ± 0.17 h^–1^ vs. PLs: 0.23 ± 0.13 h^–1^, *p* ≤ 0.05, Figure 3D). The time range that covered the dynamic increase in ADC for all samples in the NPL group and the PL group was 3.19 and 6.53 h, respectively. All parameters obtained from model fitting were averaged for NPL and PL groups and are summarized in Table 4.

**TABLE 4:** Summary of parameters from model fitting of DWI results averaged for non-perfused liver (NPL) and perfused liver (PL) samples.

	ADC_0_—initial value in 10^–3^ mm^2^/s	T—onset time in h	*K*—rate constant in h^–1^	ADCp—plateau in 10^–3^ mm^2^/s
NPL	0.43 ± 0.04	9.88 ± 1.29	0.36 ± 0.17	0.68 ± 0.08
PL	0.53 ± 0.07	12.59 ± 1.78	0.23 ± 0.13	0.98 ± 0.35

### Histological Evaluation

**FIGURE 4:**
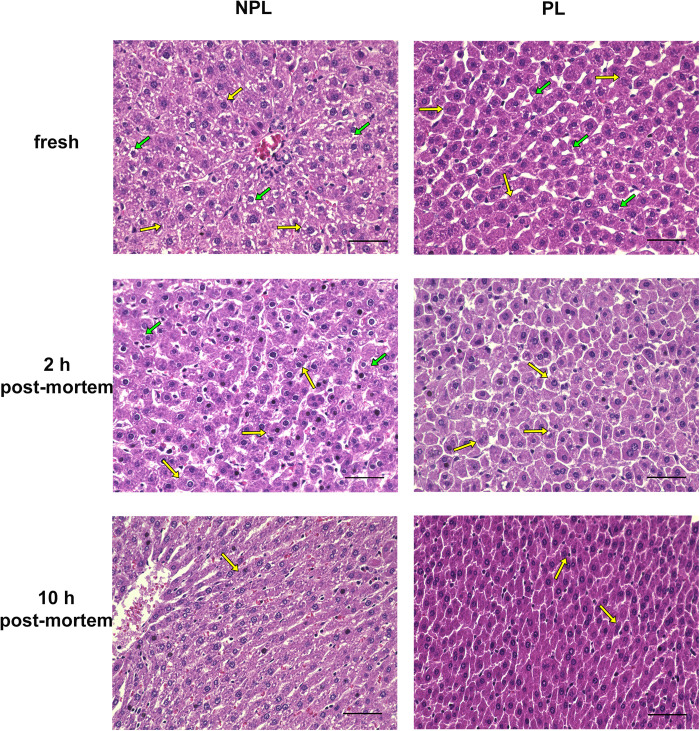
Representative H&E-stained liver sections from NPLs and PLs collected immediately after death (fresh), 2 h post-mortem, and 10 h post-mortem. Scale bars correspond to 50 μm. NPL, non-perfused liver; PL, perfused liver. Arrows indicate examples for: glycogen (green) and cell wall (yellow).

Representative microscopic images of H&E-stained liver sections from PLs and NPLs at three time points are shown in Figure 4. The most prominent histological features of these samples can be described as follows: fresh NPL samples commonly displayed structural integrity with polygonal hepatocytes tightly organized in trabeculae. Additionally, a high amount of glycogen was present, seen as white spaces inside the cytoplasm of hepatocytes. Conversely, hepatocytes in fresh PL samples tended to appear roundish rather than polygonal. Although the trabecular arrangement was still visible, some hepatocytes already demonstrated detachment from the rest, resulting in a seemingly loosened packing. Furthermore, there was almost no glycogen visible in the fresh PL sample.

At 2 h pm, the histological appearance of the NPL sample was very similar to that of the fresh PL sample as described above, showing roundish hepatocytes which were loosely packed with no visible presence of glycogen. Comparatively in the PL sample, roundish hepatocytes displayed larger-scale separation and dissociation with almost no visible hepatocyte clusters. At this time point, the nuclei appeared hyperchromatic in both NPL and PL samples, while the nuclei in the NPL sample additionally showed a slight size reduction.

At 10 h pm, the most prominent changes appearing in both the NPL and PL samples affected the shape and size of hepatocytes. In both groups, the formerly round hepatocytes observed at 2 h pm became elongated, taking on an ellipsoidal shape. Overall size of hepatocytes appeared to be smaller compared to 2 h pm. Additionally, cell walls appeared to become less distinct, making it harder to visually separate hepatocytes from one another. Furthermore, nuclei became smaller and exhibited signs of hyperchromasia and pyknosis. There was even evidence of karyorrhexis in the NPL sample. All histological findings are summarized in Table 2.

**FIGURE 5:**
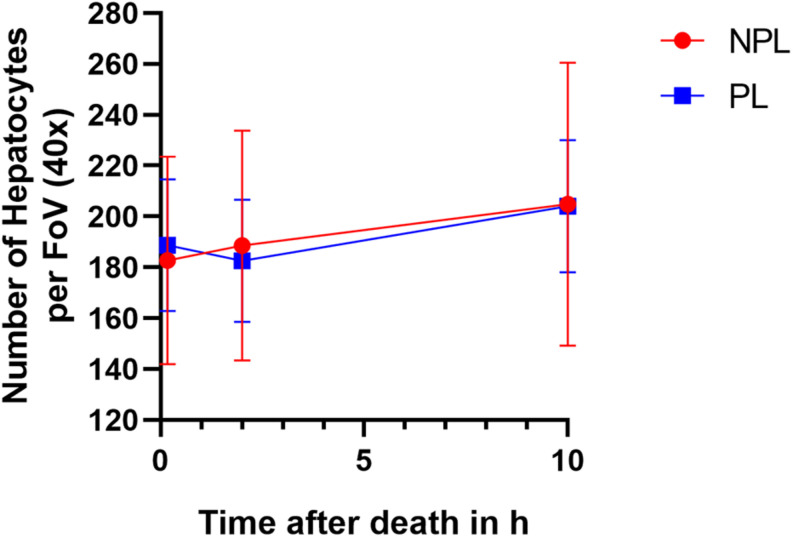
Mean with SD of numbers of hepatocytes per field of view (FoV) counted at 40x optical field at three time points (fresh, 2 h post-mortem, and 10 h post-mortem) for both NPLs (*n* = 7) and PLs (*n* = 7). For each animal, two images were analyzed. NPL: non-perfused liver; PL: perfused liver.

Hepatocytes per FoV at 40x magnification were counted at all three time points for both NPLs and PLs, and counts were further analyzed using the mixed ANOVA method. We found that the difference in cell counts between the NPL and PL groups was solely influenced by the time after death (*p* ≤ 0.01) with no significant effect of perfusion status (*p* = 0.98, Figure 5). Mean cell counts for the two groups at the different time points were as follows: fresh, NPLs: 183 ± 41 vs. PLs: 189 ± 26; 2 h pm, NPLs: 189 ± 45 vs. PLs: 183 ± 24; 10 h pm, NPLs: 205 ± 56 vs. PLs: 204 ± 26.

## Discussion

In this study, we investigated longitudinal variation of two biophysical properties, water diffusion and viscoelasticity, of perfused (PLs) and non-perfused (NPLs) livers over a long post-mortem (pm) interval with a high sampling rate. Comparison of NPLs and PLs consistently revealed higher stiffness, better wave penetration, and lower water diffusivity in NPLs for all measurements. Furthermore, we observed a significant dependency of all imaging parameters in both groups on the time after death. These findings are summarized in Table 2 and will be briefly discussed in the following paragraphs.

In both NPLs and PLs, the initial phase (Phase I) from immediately after death up to 2 h pm was characterized by the biological pattern of ischemia followed by hypoxia and anoxia, ultimately resulting in cytotoxic edema and hepatocytes swelling (Riede et al., 1976). It appears as if the enlarged hepatocytes created intracellular pressure and mechanical resistance (Bertalan et al., 2020) and formed a tightly packed and compact structure which mechanically manifested as increased stiffness and lower viscosity (i.e., higher wave penetration rate *a*). Furthermore, it is known from the literature that water mobility is restricted by cell walls, leading to diminished water diffusivity when extracellular water is shifted into the intracellular space (Le Bihan and Iima, 2015) e.g., during formation of cytotoxic edema. A similar finding of reduced ADC and increased stiffness was reported after induction of hypoxic-anoxic injury in the mouse brain (Bertalan et al., 2020). Arthurs et al. (2015) also observed a decrease in ADC values in the post-mortem liver. In addition to cytotoxic edema, blood coagulation could also contribute to higher stiffness and restricted water diffusivity (Berg, 1950; Clark et al., 1996; García-Manzano et al., 2001). The effect of coagulation should be more pronounced in NPLs than PLs; however, residual blood in PLs due to incomplete perfusion might also contribute to stiffening in NPLs. It is also worth mentioning that based on literature data, blood remains mostly fluid in the main vessels after sudden death (Mole, 1948; Berg, 1950; Haba et al., 1963; Takeichi et al., 1984, 1985, 1986; Jackowski et al., 2006; El alfy and Elhadidy, 2018), such as decapitation used in our study for the NPLs.

After the prominent changes observed in the initial phase, we identified a stable phase extending up to 10 h pm that was characterized by fairly small changes in stiffness, wave penetration and water diffusivity in both NPLs and PLs. In this phase, there were no biophysical signs of major disruption of tissue integrity or cell breakdown.

After the stable phase, the penetration rate displayed a visible time dependency from 10 h to 15 h pm in both NPL and PL groups. Comparing histological images acquired at 2 h and 10 h pm, we suspected that the increase in penetration rate was a result of changes in cell shape with subsequent alteration in cell packing and reordering of the tissue lattice. In comparison to the fresh state, which was characterized by hepatocytes arranged in a tightly packed pattern, gradual elongation of hepatocytes over time seemed to create a more parallel-oriented packing arrangement. This arrangement of elongated hepatocytes appears to reduce intercellular friction, thus facilitating wave propagation. Similar to wave penetration rate, a significant increase in water diffusivity, indicating diminished restriction of water mobility, was observed from 10 h after death until the end of measurement. Considering that no decrease in stiffness was seen and cell walls were still visible in the histological images at 10 h pm, we hypothesize that the observed increase in water diffusivity is largely due to a gradual increase in cell membrane permeability rather than cell collapse and autolysis. Increased membrane permeability with a loss of membrane integrity is usually a process of tissue degradation which is followed by cell shrinkage and cell death (Majno et al., 1960; Kimura and Abe, 1994; Milroy, 1999; Yoon et al., 2002; Malhi et al., 2006; Miller and Zachary, 2017; Yahia et al., 2018). Diffusivity started to increase earlier in NPLs, indicating faster tissue degradation in NPLs than PLs. Death induced anaerobic glycogenolysis during ischemia and anoxia produces a high amount of lactate, which leads to an acidic environment that is toxic to cells (Shima, 1922; Popper and Wozasek, 1932; Hertz, 1933; Nunley et al., 1973; Calder and Geddes, 1990; Donaldson and Lamont, 2013). Calder and Geddes (1990) reported the highest glycogen and lactate concentration in *ex vivo* livers of Wistar rats 30 min after death. Accumulation of lactate occurred in both NPLs and PLs in our study; however, due to perfusion, the high lactate-containing blood was replaced by PBS in PL samples, which apparently delayed the process of autolysis and tissue degeneration (Shima, 1922; Mukundan et al., 1986; George et al., 2016). We also found that the time window for observing such an increase in water diffusivity was narrower in NPLs than PLs, suggesting an inhomogeneity of residual blood remaining in PL vessels and leading to more heterogeneous tissue degradation in PLs. Additionally, the increase in cell membrane permeability and successive release of apoptogenic proteins and other small solutes might also function as intercellular lubricant that reduced friction and promoted wave propagation, as mentioned earlier. Since membrane permeability increases during tissue degradation, Phase III and the extended Phase III for DWI are considered to be degradation phases.

Despite the encouraging results, our study has limitations. First, no anticoagulant was used due to ethical restrictions in our study protocol. Therefore, we cannot rule out that some blood remained within the hepatic vessels. Second, the number of samples used for histological analysis was small so that the statistical power for cell counts was limited. Further studies investigating *ex vivo* tissue from other organs while controlling and varying factors such as medium osmolality, degree of cell hydration, and temperature levels are planned in order to gain deeper insights into autolysis and tissue decomposition and their biophysical manifestations.

To summarize, both MRE and DWI provided biophysical imaging parameters sensitive to structural alterations of rat livers during a cascade of biological events occurring after death. Throughout the 15-h pm interval investigated here, NPLs always showed higher stiffness, better penetration rate and lower water diffusivity than PLs. During this interval, three distinct phases were discernible - an initial phase with marked changes in biophysical properties up to 2 h pm, a stable phase up to 10 h pm, and a degradation phase characterized by significant increases in wave penetration and water diffusivity. As supported by histological results, these observed changes in biophysical parameters were closely related to variations in cell shape, cell packing patterns and cell wall integrity. Although obtained ex vivo, our results shed light on the individual contributions of vascular components and cellular integrity to liver biomechanical properties that can be quantified by elastography. In future applications of elastography, our results might support the interpretation of the biophysical signature related to liver tissue degradation.

## Data Availability Statement

The raw data supporting the conclusions of this article will be made available by the authors, without undue reservation.

## Ethics Statement

The animal study was reviewed and approved by the Landesamt für Gesundheit und Soziales Berlin.

## Author Contributions

KG: conceptualization, data acquisition, investigation, formal analysis, data curation, writing—original draft, editing, and visualization. HT: software, validation, and writing—review and editing. AK: histological investigation, and writing, review and editing. SA, AH, ES, NB, and H-GH: writing—review and editing. JB: funding acquisition, resources, and methodology. IS: conceptualization, funding acquisition, resources, methodology, project administration, supervision, critical and revision of the manuscript. JG: conceptualization, data curation, formal analysis, funding acquisition, investigation, methodology, visualization, supervision, writing—original draft, writing—review and editing, and critical revision of the manuscript. All authors fully qualify for authorship and have approved the final version of the manuscript.

## Conflict of Interest

The authors declare that the research was conducted in the absence of any commercial or financial relationships that could be construed as a potential conflict of interest.

## Publisher’s Note

All claims expressed in this article are solely those of the authors and do not necessarily represent those of their affiliated organizations, or those of the publisher, the editors and the reviewers. Any product that may be evaluated in this article, or claim that may be made by its manufacturer, is not guaranteed or endorsed by the publisher.

## Footnotes

1
www.graphpad.com
